# Mapping Geographic Access to Illinois Birthing Hospitals, 2016–2023

**DOI:** 10.5888/pcd21.240332

**Published:** 2024-12-26

**Authors:** Barbara C. Keino, Mechelle D. Claridy, Laurin Kasehagen, Jessica R. Meeker, Lauren M. Ramsey, Elizabeth J. Conrey, Amanda C. Bennett

**Affiliations:** 1 Epidemic Intelligence Service, Public Health Infrastructure Center, Division of Workforce Development, Centers for Disease Control and Prevention, Atlanta, Georgia; 2 HIV Surveillance Branch, Division of HIV Prevention, National Center for HIV, Viral Hepatitis, STD, and TB Prevention, Centers for Disease Control and Prevention, Atlanta, Georgia; 3 Maternal and Child Health Epidemiology Program, Field Support Branch, Division of Reproductive Health, National Center for Chronic Disease Prevention and Health Promotion, Centers for Disease Control and Prevention, Atlanta, Georgia; 4 Emergency Preparedness and Response Team, Field Support Branch, Division of Reproductive Health, National Center for Chronic Disease Prevention and Health Promotion, Centers for Disease Control and Prevention, Atlanta, Georgia; 5 US Public Health Service, Rockville, Maryland

**Figure:**
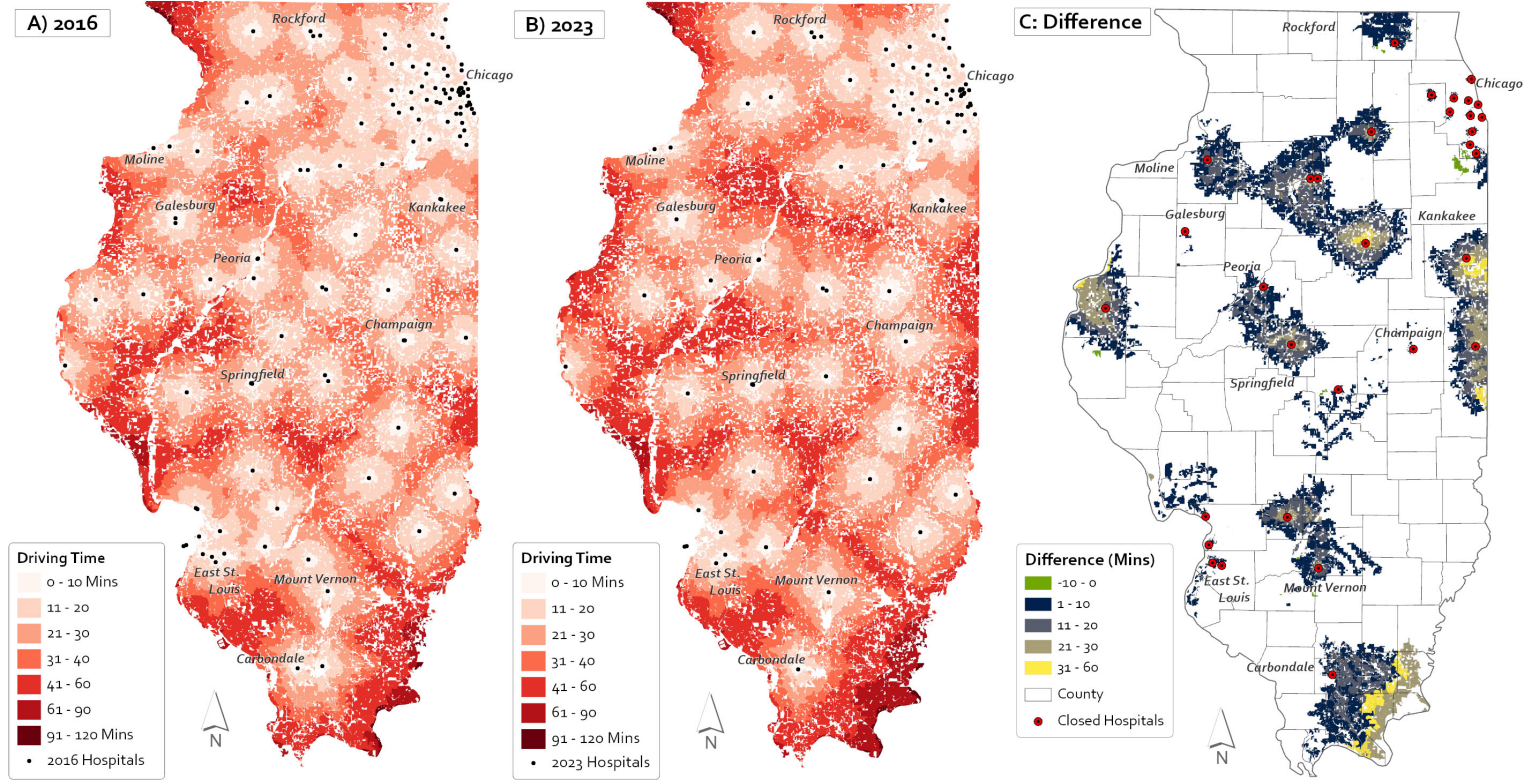
Three maps depict driving time from Illinois census blocks to the nearest birthing hospital in 2016 (Map A) and 2023 (Map B). Driving time to the nearest birthing center increased near hospital closures, particularly in the east and southeast, near Kankakee and Carbondale (Map C). Source: Illinois Department of Public Health, US. Census Bureau.

## Purpose

Timely access to quality obstetric care is a critical component in promoting maternal health and positive birth outcomes (1). Access to risk-appropriate care, or care in facilities equipped with necessary personnel and services, is critical for optimizing obstetric and neonatal outcomes (2). The closure of hospital-based obstetric services, which include specialized resources and a specialized health care workforce, has been associated with an increase in out-of-hospital and preterm births, particularly in rural areas (3–5). Additionally, longer travel distances may delay or disrupt receipt of prenatal care, impede specialized care for patients with high-risk conditions, and adversely affect birth outcomes (6).

The objectives of our study were to assess the spatial and temporal changes in geographic access to Illinois birthing hospitals from 2016 to 2023 for women of reproductive age (15 y to 49 y) residing in Illinois. Additionally, we illustrate the use of novel methods to estimate geographic access, by using isochrones (areas that represent equal travel time from a central location) to calculate driving time and census blocks, the smallest US Census spatial unit, to estimate access to birthing hospitals for women of reproductive age. This approach captures drive time estimates for all populated census blocks in Illinois, in contrast to census tract or county-level centroid analyses, which may obscure travel times for populations not residing near the geographic or population center of these larger spatial units.

## Data and Methods

We obtained Illinois birthing hospital addresses and closures from 2016, the first year of data collection, to 2023 from the Illinois Department of Public Health. We used ESRI ArcGIS (Esri) to geocode the locations of these hospitals and used an isochrone-based approach to calculate drive times to the hospitals in 10-minute increments from 0 to 40 minutes, followed by increments of 40 to 60 minutes, 60 to 90 minutes, and 90 to 120 minutes.

Drive-time isochrones represent the area accessible within a specified driving time to or from a particular point of interest, such as a hospital. Compared with the straight-line distance measures often used in birthing hospital access literature (7,8), drive-time isochrones provide a more accurate estimate of geographic access by accounting for travel routes, speed limits, and traffic patterns. This method considers the real-world complexities of travel, such as road network layouts and obstacles, resulting in a more realistic measure of how long it takes to reach a specific location, such as a hospital. Accuracy is crucial when assessing geographic access to health care services because travel time may affect health outcomes (9).

We obtained US Census block polygons and demographic information (age, sex, rural or urban place of residence) from the Integrated Public Use Microdata Series National Historical Geographic Information System (10). The US Census uses census blocks, the smallest geographic sampling unit, to tabulate decennial data that are then aggregated into larger spatial units such as census tracts and counties. In Illinois, census blocks average 0.16 square miles with an average population of 35 people. In comparison, census tracts average 17.3 square miles with 3,929 people, and counties average 552.4 square miles with 126,107 people.

To assess geographic access to Illinois birthing hospitals for women of reproductive age, we converted census block polygons to geographic centroids and joined them with isochrone polygons to determine the drive-time increment for each block. We visualized the spatiotemporal patterns of geographic access to birthing hospitals in maps for 2016 (Map A) and 2023 (Map B) and the driving time difference between these years (Map C). Additionally, we tabulated a summary of the proportion of women residing within 10, 30, 60, 90, and 120 minutes of a birthing hospital in 2016 and 2023 and the change over time, stratified by rural or urban residence. To generate an estimate of the birthing population that may be affected by birthing hospital closures, we assumed that the population of women of reproductive age remained static from 2016 through 2023.

## Highlights

From 2016 to 2023, the number of birthing hospitals in Illinois decreased from 118 to 86, affecting geographic accessibility for women of reproductive age residing in Illinois (Map C). Women mostly resided in urban census blocks (89.4%, n = 2,635,775) compared with rural census blocks (10.6%, n = 313,273). In 2016, 76.5% (n = 239,654) of women in rural census blocks lived within 30 minutes of a birthing hospital, compared with 99.1% (n = 2,612,053) of women in urban census blocks. By 2023, these percentages decreased to 65.4% (n = 204,881) for rural women and 98.0% (n = 2,583,060) for urban women.

These findings highlight a decline in geographic access to birthing hospitals in Illinois from 2016 to 2023, especially for women of reproductive age in rural areas, where 11.1% (n = 34,773) of women were no longer within a 30-minute drive, compared with 1.1% (n = 28,993) in urban areas. Although most women of reproductive age live in urban areas, rural women experienced a greater decline in geographic access from 2016 to 2023, leading to longer travel times and potentially delaying essential obstetric care, which may exacerbate rural–urban maternal health disparities.

## Action

The maps from our analysis depict the increase in driving time to the nearest birthing hospital resulting from birthing hospital closures, particularly in east and southeast Illinois, resulting in decreased geographic access in rural areas. Equitable access is essential for achieving positive and equitable maternal and infant health outcomes. Access to timely care may play a role in the disparities that exist in maternal health outcomes by rurality (11). Strategies could address gaps in access to high-quality obstetric health care in rural areas. The National Rural Health Association recommends strategies such as obstetric training and simulations for rural health care providers in hospital emergency departments, telemedicine consultation with regional perinatal centers, improved equipment and consultation resources for emergency medical services, and support of a doula workforce in rural communities to reduce pregnancy complications (12). The American College of Obstetricians and Gynecologists describes the importance of regionalized perinatal centers for providing rural hospitals with ready access to consultation, referral, and outreach education, and in establishing interhospital agreements for the timely transport of pregnant patients (13).
